# Cross‐reactivity between tick and wasp venom can contribute to frequent wasp sensitization in patients with the α‐Gal syndrome

**DOI:** 10.1002/clt2.12113

**Published:** 2022-01-17

**Authors:** Mensiena B. G. Kiewiet, Marija Perusko, Jeanette Grundström, Carl Hamsten, Maria Starkhammar, Danijela Apostolovic, Marianne van Hage

**Affiliations:** ^1^ Division of Immunology and Allergy Department of Medicine Solna Karolinska Institutet and University Hospital Stockholm Sweden; ^2^ Department of Internal Medicine Södersjukhuset Stockholm Sweden

**Keywords:** α‐Gal syndrome, cross‐reactivity, *Ixodes ricinus*, red meat allergy, tick, *Vespula vulgaris*, wasp

## Abstract

**Background:**

α‐Gal syndrome (AGS) is a food allergy with severe delayed allergic reactions, mediated by IgE‐reactivity to galactose‐α1,3‐galactose (α‐Gal). AGS is strongly associated with tick bites. An increased incidence of venom sensitization has been found in AGS patients. Here, we evaluated the frequency of wasp sensitization in Swedish AGS patients and the possible cross‐reactivity between wasp venom and tick proteins.

**Methods:**

Sera from 136 Swedish AGS patients and 29 wasp‐positive non‐AGS control sera were analyzed for IgE‐reactivity against wasp venom (*Vespula* spp.), the European tick *Ixodes ricinus* (Streptavidin ImmunoCAP), α‐Gal and total IgE by ImmunoCAP. The presence of α‐Gal on wasp venom proteins (*Vespula vulgaris*) was investigated by western blot (WB), and possible cross‐reactivity between wasp venom and tick proteins by enzyme‐linked immunosorbent assay and WB. Involvement of cross‐reactive carbohydrate domains (CCDs) was also assessed.

**Results:**

Wasp sensitization was present in 54% of AGS patients, although the IgE levels were low. Wasp sensitized patients had higher IgE levels to α‐Gal and total IgE levels compared to non‐wasp sensitized AGS patients. α‐Gal was not detected in wasp venom, but cross‐reactivity between wasp and tick proteins was demonstrated which was not dependent on CCDs. The same cross‐reactivity was also observed in the control sera. Furthermore, 17 putative cross‐reactive peptides were identified using an *in silico* approach.

**Conclusions:**

For the first time, cross‐reactivity between wasp venom and tick proteins has been described. This may be a reason why the majority of Swedish AGS patients, who have all been tick bitten, are also sensitized against wasp.

## 1 BACKGROUND

IgE antibodies against the carbohydrate epitope galactose‐α1,3‐ galactose (α‐Gal) are the cause of a novel type of food allergy, the α‐Gal syndrome (AGS), which is characterized by severe allergic reactions, for example, angioedema, urticaria, and anaphylaxis, 2–7 h after the consumption of mammalian products. Interestingly, the disease onset is strongly associated with tick bites.^1^ Many tick species have been found to contain α‐Gal,^2^, ^3^ and the carbohydrate is thought to be transmitted to the host during bites. Furthermore, multiple consecutive tick bites have been observed to cause an increase of α‐Gal‐specific IgE levels,^4^ while avoiding tick bites results in a decrease of α‐ Gal‐specific IgE.^5^

The observed relation between tick bites and AGS has raised the question whether other biting or stinging arthropods may be associated with AGS as well. Until now, available data is limited. In the US, larvae of the Trombiculidae mites, or “chiggers,” were suspected to have caused α‐Gal sensitization in a few cases.^6^

Another class of insects that have been linked to AGS are the hymenoptera, including bees and wasps. Venom allergy has been found in 24% (12/50) of meat allergic patients from a Turkish case study, and honey bee venom allergy was the most frequent.^7^ A study from the US showed that AGS patients were 5 times more likely to be sensitized to hymenoptera venom compared to controls.^8^ Of all investigated venoms, wasp‐specific IgE was most common. Several factors may explain this observation. First, the prevalence of α‐Gal sensitization is known to be relatively high among people spending much time outdoors.^9^, ^10^ Being outdoors not only increases the risk of tick bites leading to α‐Gal sensitization, but also of wasp stings.^11^, ^12^ Secondly, certain immunological characteristics might contribute to the host being more prone to sensitization against tick and wasp. For example, atopy has been associated with wasp sensitization^13^ as well as with tick‐induced AGS.^14^ Finally, molecular similarities between tick and wasp proteins might lead to cross‐reactivity. Wasp proteins are known to cross‐react with a range of other hymenoptera^15^ as well as to taxonomically more distant species.^16^ However, possible cross‐reactivity with tick proteins has not been studied so far.

In this study, we explored wasp sensitization in AGS patients on a molecular level. We analyzed wasp‐specific IgE levels in a Swedish AGS cohort. Furthermore, we investigated the presence of α‐Gal on wasp venom proteins (*Vespula* spp.) and possible cross‐reactivity with tick proteins (*Ixodes ricinus*), in order to examine if properties of wasp venom proteins could be involved in the increased wasp sensitization in AGS patients. Finally, we elucidated if the same results could be observed in a wasp‐positive, non‐AGS control group.

## 2 MATERIALS AND METHODS

### 2.1 Patient and control sera

Sera of 136 AGS patients with IgE to α‐Gal and allergic symptoms after mammalian meat intake were included. All patients provided information regarding AGS symptoms, tick exposure and presence of other allergies by filling in a questionnaire and an in‐depth interview conducted by the same allergologist with many years of experience in food allergy and AGS. The majority of these patients have previously been carefully characterized.^14^ To compare, a group of 29 wasp‐positive sera (non‐AGS controls) were randomly selected from the biobank at the Department of Clinical Immunology, Karolinska University Hospital (Stockholm, Sweden; Table S1). Allergen‐specific IgE levels against α‐Gal (bovine thyroglobulin [bTG], o215), wasp (*Vespula vulgaris,* i3), tick (*I. ricinus*), and total IgE were determined for all sera by ImmunoCAP (Phadia AB/Thermo Fisher Scientific). IgE antibodies against tick were measured by coupling biotinylated tick protein extract to Streptavidin ImmunoCAP, (o212, Phadia AB/Thermo Fisher Scientific) as previously described.^2^ The cut‐off for allergen‐specific IgE was ≥0.1 kU_A_/L. A selection of sera, both from AGS patients and controls, were either used individually or as serum pools in experiments described below (Table 1). The study was approved by the Swedish ethical review authority (Ethical permit No 2011/1604‐31/2, 2016/1348‐32, 2018/2483‐32, 2020‐01686) and performed in accordance with the declaration of Helsinki. All patients gave their written informed consent.

**TABLE 1:** Characteristics of serum pools

Serum	Number of sera	Wasp IgE (kU_A_/L)	Tick IgE (kU_A_/L)	α‐Gal IgE (kU_A_/L)
AGS patients				
(1) Dominantly tick‐positive	5	0.54	5.2	30
(2) Dominantly wasp‐positive used in WB and ImmunoCAP	3	17	17	62
(3) Wasp‐positive used in ELISA	6	11.4	5.2	34.0
Wasp‐positive controls				
(4) Wasp‐ and tick‐positive	11	11	1.2	<0.10
(5) Only wasp‐positive	6	14	<0.10	<0.10
Individual AGS patient sera used in ELISA				
(1)		38.0	54.0	76.0
(2)		5.8	3.3	4.4
(3)		20.0	7.8	100.0
(4)		2.5	0.11	7.0
(5)		2.2	4.2	54.0

Abbreviation: ELISA, enzyme‒linked immunosorbent assay; WB, western blot.

### 2.2 Wasp venom and tick protein extract

Wasp venom (*Vespula vulgaris* and *Vespula germanica* mix) was purchased from Citeq Biologics. The lyophilized product was reconstituted in Phosphate‐buffered saline (PBS), and the protein concentration was determined using the Bradford method.^17^ Protein extracts from pathogen‐free *I. ricinus* adults (IS Insect Service GmbH) were prepared as previously described.^18^ The protein concentration was determined using the bicinchonic acid protein assay.

### 2.3 SDS‐PAGE and WB analysis

Wasp venom and tick protein extract (each 30 μg per lane) were separated under reducing conditions on sodium dodecyl sulphate–polyacrylamide gel electrophoresis (SDS‐PAGE) at TGX™ gradient precast gels (any kDa, Bio‐Rad Laboratories) using a Mini Protean Cell II system (Bio‐Rad Laboratories). Protein bands were stained with Coomassie Brilliant Blue. For western blot (WB), proteins were transferred to polyvinylidene difluoride membranes (0.2 μm pore size) using a Bio‐Rad Turbo system. Membranes were blocked with 1% human serum albumin in phosphate buffered saline containing 0.05% Tween for 2 h at room temperature (RT).

To detect α‐Gal carrying proteins, the membrane was incubated with 1:7500 dilution of chicken anti‐α‐Gal single‐chain antibody variable‐region fragment (a kind gift from the National University of Ireland, Galway, Republic of Ireland)^19^ labeled with hemagglutinin tag for 2 h at RT. Next, the membrane was incubated with 0.25 μg/ml mouse monoclonal anti‐hemagglutinin antibody (Cat. No. H3663, Sigma Aldrich) for 1 h, followed by goat anti‐mouse IgG labeled with alkaline phosphatase (AP; 1:1000, Jackson ImmunoResearch Laboratories). AP Conjugate Substrate Kit (Bio‐Rad Laboratories) was used for visualization.

IgE binding to wasp venom and tick proteins was tested by incubating the membrane with serum pools from AGS patients (Pool 1 and 2; Table 1) or wasp‐positive controls (Pool 4 and 5; Table 1) overnight with agitation. For inhibition of IgE binding, the serum pools were preincubated with 100 μg/ml of either wasp venom or tick protein extract for 1 h prior to addition to the membrane. To assess the role of cross‐reactive carbohydrate determinant (CCD)‐specific IgE, serum was preincubated with a commercial CCD‐inhibitor (anti‐CCD absorbant, EuroImmun) following the manufacturer's instructions before addition to the membrane. Bound IgE was detected with mouse anti‐human IgE labeled with horseradish peroxidase (1:2000, Abcam) for 1 h at RT. Visualization was performed with luminol and H_2_O_2_ substrate (GE Healthcare) on a ChemiDoc instrument (Bio‐Rad Laboratories).

### 2.4 Inhibition ImmunoCAP

In order to investigate if α‐Gal‐specific IgE is involved in IgE recognition of wasp venom, a dominantly wasp‐positive AGS patient serum pool (Pool 2; Table 1) was preincubated with 1 mg/ml α‐Gal containing bTG at RT for 1 h, after which the wasp‐specific IgE was measured using ImmunoCAP following the manufacturer's instructions (Phadia/Thermo Fisher Scientific). The same serum pool incubated with PBS served as a control.

### 2.5 Inhibition ELISA

Inhibition enzyme‐linked immunosorbent assay (ELISA) was used to investigate the inhibitory capacity of wasp venom and tick protein extract on the IgE binding to tick and wasp venom protein, respectively. Microtiter plates were coated with 10 μg/ml wasp venom or tick protein extract in coating buffer (50 mM sodium bicarbonate buffer pH 9.6) overnight at +4°C. Serum pools (Pool 1 and 3; Table 1) were pre‐incubated with 2‐fold serial dilutions of wasp venom or tick protein extract (6.25–400 μg/ml), while sera from individual subjects were preincubated with 400 μg/ml wasp venom or tick protein extract or 1 mg/ml bTG, both for 1 h at RT. The remaining IgE binding was detected with mouse anti‐human IgE labeled with horseradish peroxidase (1:3000, Abcam). Visualization was performed as described above. The optical density was read at 450 nm. Inhibition of IgE binding was presented as the percentage of the total IgE binding.

### 2.6 In silico sequence similarity search

In order to identify cross‐reactive epitopes, sequence similarity between tick hemelipoglycoprotein (A0A0D3RJ94) and wasp vitellogenin (G8IIT0) was assessed by calculating the property distance (PD) value using the peptide similarity tool of the Structural database of allergenic proteins (SDAP, http://Fermi.utmb.edu/SDAP/sdap_pdi.html).^20^ Sequential peptides of 10 amino acids, overlapping by seven (three‐offset), in the sequence of tick hemelipoglycoprotein were aligned with the amino acid sequence of G8IIT0. Since peptides with PD values below 7 have been described before to be more likely to bind IgE than those with higher PD values,^21^ peptides with a PD value < 7 were considered to be possible cross‐reactive peptides.

### 2.7 Statistics

Data were analyzed using GraphPad Prism software, version 8. Mann‐Whitney U‐tests were used to compare sensitized and non‐sensitized AGS patients. Spearman's rank correlation was used to calculate the strength of an association between two variables. A *p*‐value < 0.05 was considered significant.

## 3 RESULTS

### 3.1 IgE against wasp venom is common among α‐Gal syndrome patients

**FIGURE 1:**
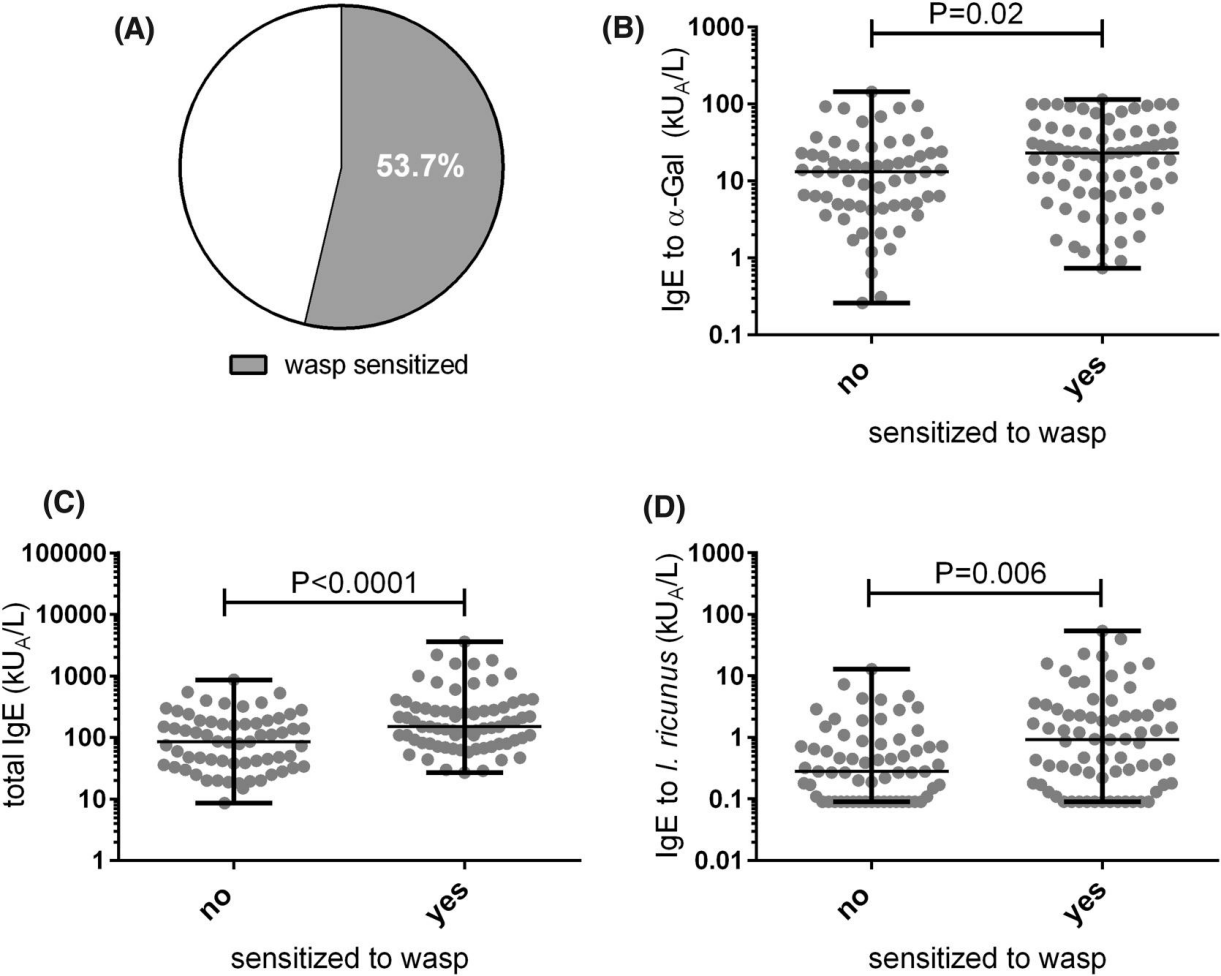
Wasp sensitization in α‐Gal syndrome (AGS) patients. (A) Percentage of wasp‐sensitized AGS patients. (B) IgE levels against α‐Gal, (C) total IgE levels, and (D) IgE levels against *Ixodes ricinus* in wasp‐sensitized AGS patients compared to non‐sensitized AGS patients

Of the 136 included AGS patients, 73 (53.7%) had IgE reactivity against wasp (Figure 1A), with a median IgE level of 0.54 kU_A_/L (range 0.1–38 kU_A_/L). These wasp‐sensitized AGS patients showed significantly higher levels of α‐Gal‐specific IgE and total IgE compared to non‐wasp sensitized AGS patients (Figure 1B,C). A poor but significant correlation was found between α‐Gal‐specific and wasp‐specific IgE (*ρ* = 0.21, *p* < 0.01, data not shown).

More than 80% of the 73 wasp‐positive AGS patients (*n* = 60) had IgE reactivity to tick. Of these double‐positive patients, 41 had higher tick‐specific than wasp‐specific IgE levels and 19 higher IgE levels to wasp than to tick. Furthermore, tick‐specific IgE levels were also significantly higher in wasp‐sensitized AGS patients compared to non‐wasp sensitized AGS patients (median 0.93 kU_A_/L; range <0.10–54.0 kU_A_/L vs. 0.28 kU_A_/L range <0.10–12.9 kU_A_/L; *p* < 0.006; Figure 1D). A low, but significant correlation was found between wasp‐specific and tick‐specific IgE levels (*ρ* = 0.29, *p* < 0.0005, data not shown).

We compared the results with 29 wasp‐positive non‐AGS sera (median 4.8 kU_A_/L, range 0.48–100 kU_A_/L). Eight sera were found to be tick‐positive (27.5%, median 8.2 kU_A_/L, range 0.16–20 kU_A_/L; Table 1). Of these, five had higher levels to wasp than to tick, while three had higher levels to tick than to wasp. Five sera were positive for α‐Gal, but the levels were low (median 0.30 kU_A_/L, range 0.14.–1.3 kU_A_/L). Four of these sera were both α‐Gal‐ and tick‐positive.

### 3.2 No α‐Gal bearing proteins were detected in wasp venom

**FIGURE 2:**
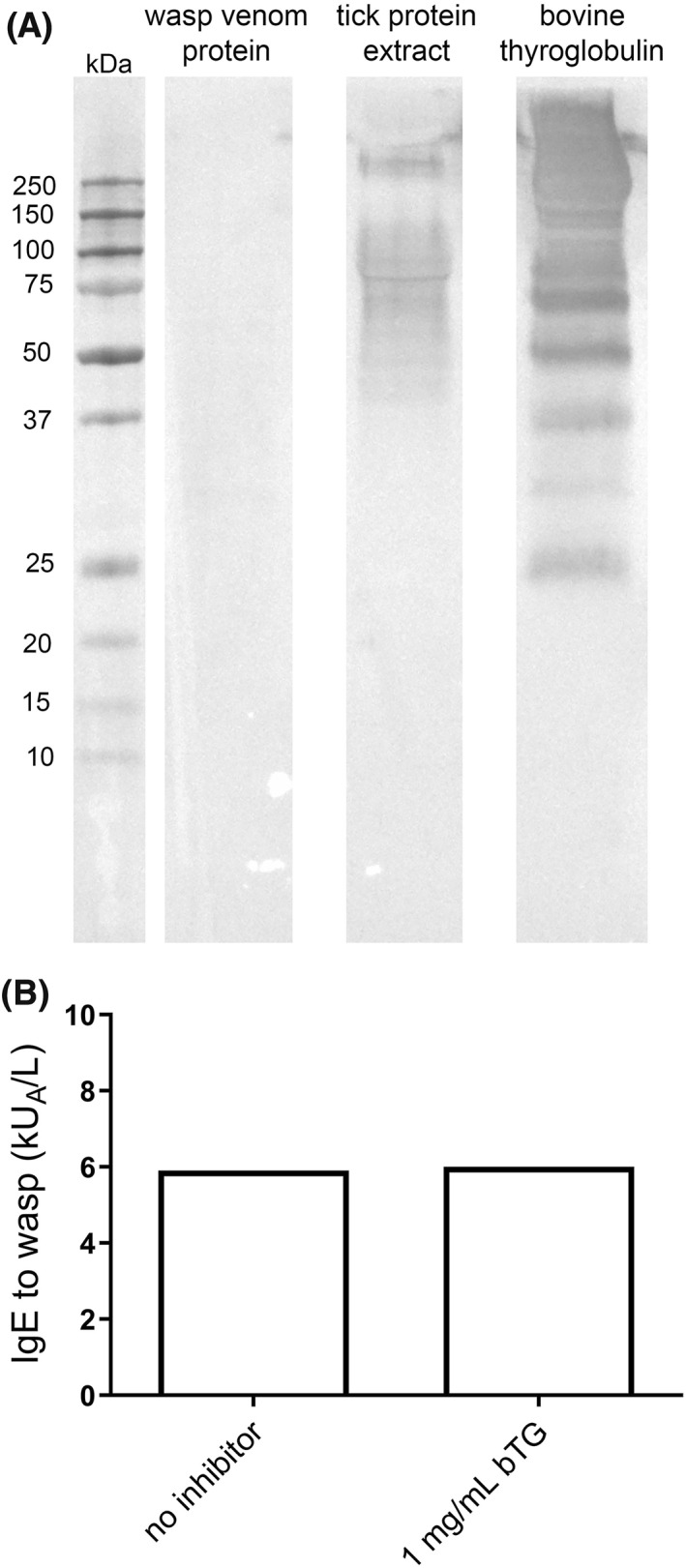
Detection of the α‐Gal epitope. (A) Western blot detection of the α‐Gal epitope in wasp venom protein and tick protein extract. Bovine thyroglobulin (bTG) was used as a positive control. (B) Inhibition of IgE binding to wasp proteins by bTG. A wasp‐positive α‐Gal syndrome serum pool (Pool 2) was preincubated with 1 mg/ml bTG before IgE levels to *Vespula vulgaris* were determined using ImmunoCAP (i3)

When analyzing the presence of α‐Gal on wasp and tick proteins by WB using an anti‐α‐Gal antibody, no α‐Gal bearing proteins in wasp venom were detected (Figure 2A). However, a range of α‐Gal‐containing proteins, both at >250 kDa, and between 150 and 37 kDa, were detected in the tick protein extract (Figure 2A). Bovine thyroglobulin, used as a positive control, showed strong α‐Gal‐specific binding. When a wasp‐positive AGS serum pool (Pool 2, Table 1) was preincubated with 1 mg/ml bTG, the wasp‐specific IgE levels, measured by ImmunoCAP, were not affected, providing evidence that α‐Gal‐specific IgE is not involved in IgE recognition of wasp venom (Figure 2B).

### 3.3 Tick protein extract inhibits IgE binding to wasp proteins in α‐Gal syndrome patients and controls

**FIGURE 3:**
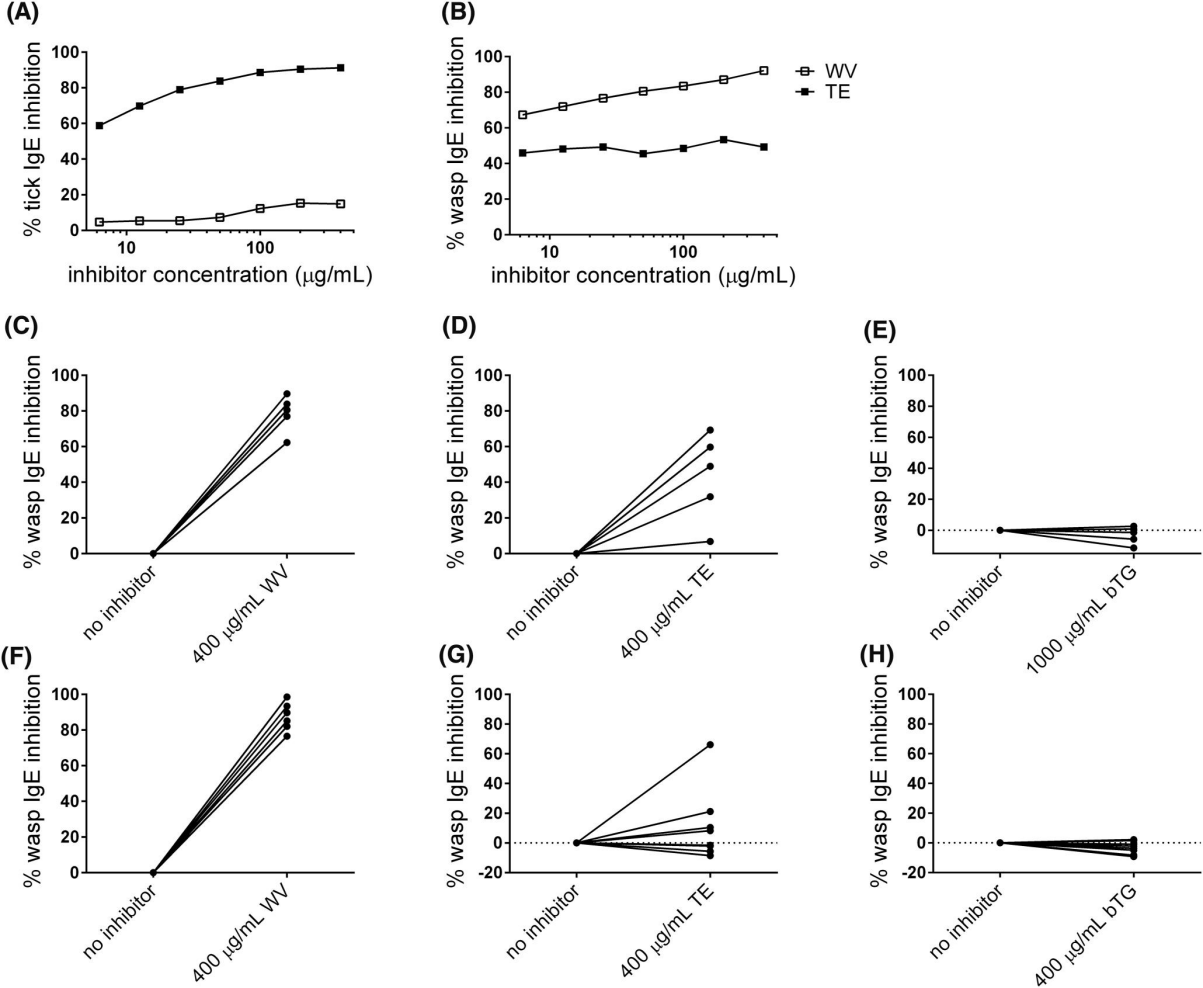
Cross‐reactivity of wasp venom and tick protein extracts assessed in enzyme‐linked immunosorbent assay (ELISA). (A) Inhibition of IgE binding to tick protein by wasp venom. A dominantly tick‐positive α‐Gal syndrome (AGS) patient serum pool (Pool 1) was preincubated with wasp venom extract and the remaining binding to tick protein was measured by ELISA. Tick protein extract was used as a positive control. (B) Inhibition of IgE binding to wasp venom proteins by tick protein extract. A wasp‐ and tick positive AGS patient serum pool (Pool 3) was preincubated with tick protein extract and the remaining binding to wasp protein was measured by ELISA. Wasp venom was used as a positive control (C–E). Inhibition of IgE binding of individual AGS patient sera to wasp venom protein by wasp venom, tick protein extract and bTG (F–H). Inhibition of IgE binding of individual wasp‐positive control sera to wasp venom protein by wasp venom, tick protein extract and bTG. Percentage inhibition of IgE binding is shown. bTG, bovine thyroglobulin; TE, tick protein extract, WV, wasp venom

Cross‐reactivity between wasp venom and tick protein extract was assessed in inhibition ELISA. Preincubation of a dominantly tick‐positive AGS patient serum pool (Pool 1, Table 1) with a range of wasp venom dilutions showed slight inhibition of the IgE‐binding to tick proteins (Figure 3A). At the highest concentration of 400 μg/ml the inhibition was less than 15%, while the homologous inhibition was almost complete. On the other hand, preincubating a wasp‐positive AGS patient serum pool (Pool 3, Table 1) with a range of tick protein dilutions showed inhibition of the IgE‐binding to wasp proteins (Figure 3B). The observed inhibition only differed slightly between different concentrations, ranging from 45.5% to a maximum of 53.4%.

Furthermore, inhibition of IgE binding to wasp by tick protein extract was investigated in five individual wasp‐positive sera from AGS patients (Table 1, IgE to wasp 2.2–38 kU_A_/L; Figure 3C–E). The homologous inhibition was high in all sera (Figure 3C, >60% inhibition). Tick protein extract (400 μg/ml) inhibited IgE binding to wasp protein in all five patients, but in a wide range, 7%–70%, indicating different levels of cross‐reactivity among individual patients (Figure 3D). For comparison, 8 individual wasp‐positive control sera were investigated in the same manner (Table 1, sera 2, 4, 8, 9, 18, 20, 22, and 27; Figure 3F–H). The homologous inhibition was high in all sera (Figure 3F, >75% inhibition). However, the inhibition with tick protein extract varied. No inhibition of the IgE binding to wasp was noted in 4 sera (Figure 3G) and in the other 4 sera the inhibition varied largely (8.2%–66%). Tick extract exerted strong inhibition of IgE binding to wasp protein in only one serum. This was the only serum with higher IgE levels to tick than to wasp (serum 18; Table 1). As expected, both in AGS patient and control sera, bTG did not induce inhibition of wasp‐specific IgE (Figures 3E,H).

### 3.4 Specific cross‐reactive proteins could be identified in tick and wasp proteins

**FIGURE 4:**
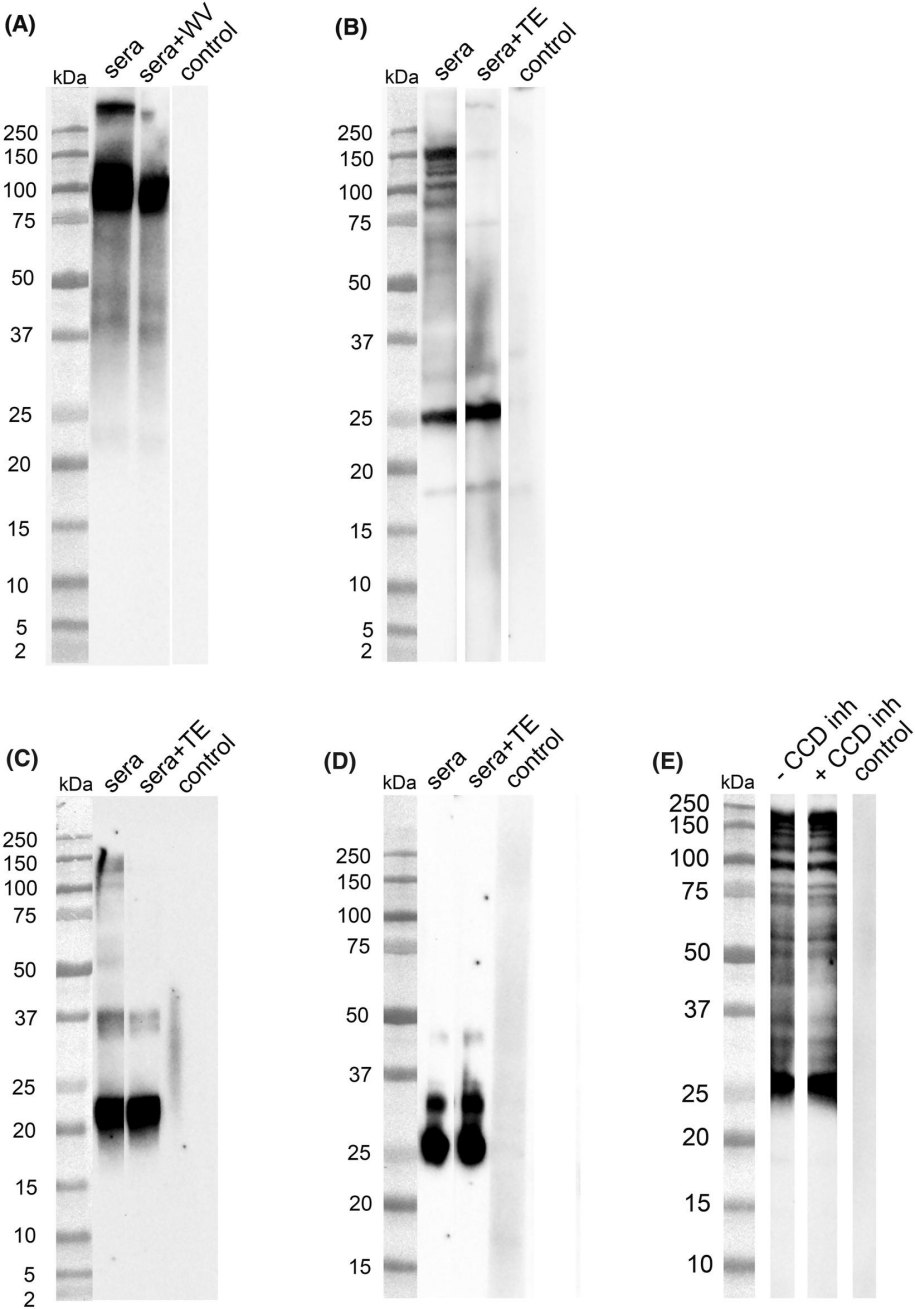
Cross‐reactivity of wasp venom and tick protein extract assessed in western blot. (A) IgE binding to tick protein of a dominantly wasp‐positive α‐Gal syndrome (AGS) patient serum pool (Pool 2) with or without preincubation with 100 μg/ml wasp venom. (B) IgE binding to wasp venom protein of a dominantly tick‐positive AGS patient serum pool (Pool 1) with or without preincubation with 100 μg/ml tick protein extract. (C) IgE binding to wasp protein of a wasp‐ and tick‐positive serum pool of controls (Pool 4) with or without preincubation with 100 μg/ml tick extract. (D) IgE binding to wasp protein of a wasp‐positive serum pool of controls (tick‐negative, Pool 5) with or without preincubation with 100 μg/ml tick extract. (E) IgE binding to wasp venom protein of a dominantly tick‐positive AGS patient serum pool (Pool 1) with or without preincubation with a CCD‐inhibitor. Control, secondary antibody control; TE, tick protein extract; WV, wasp venom

To further investigate the IgE cross‐reactivity between tick and wasp proteins, WBs with tick and wasp proteins were probed with dominantly wasp‐ or dominantly tick‐positive serum pools from AGS patients. The dominantly wasp‐positive AGS patient serum pool (Pool 2, Table 1) identified tick protein bands at >250 kDa and between 150 and 100 kDa, which were inhibited by wasp venom (Figure 4A). These bands have been earlier identified as hemelipoglycoprotein (290 kDa) and its subunit in several *Ixodes* species.^22^

The dominantly tick‐positive AGS patient serum pool (Pool 1, Table 1) recognized multiple wasp venom bands between 200 and 75 kDa, which were inhibited by tick protein extract (Figure 4B). These bands have earlier been identified as vitellogenin and its subunits.^23^, ^24^ A band at ∼23 kDa, corresponding to Ves v 5 ^25^, was also observed. This band could not be inhibited by tick extract.

The IgE binding of the AGS sera was compared with wasp‐positive non‐AGS control sera. A wasp‐and tick‐positive serum pool (Pool 4, Table 1) as well as a wasp‐positive and tick‐negative serum pool (Pool 5, Table 1) recognized the wasp allergens Ves v 5 (∼23 kDa)^25^ and Ves v 1 (∼35 kDa),^26^ (Figures 4C,D). Only the wasp‐ and tick‐positive serum pool showed multiple bands between 200 and 75 kDa, which were inhibited by preincubation with tick protein extract (Figure 4C). In contrast, the wasp‐positive and tick‐negative serum pool did not recognize these bands (Figure 4D). Figure S1 shows the SDS‐PAGE protein patterns of wasp venom and tick extract, where all mentioned proteins have been indicated as well. The amount of hemelipoglycoprotein (Band 5) in tick extract was found to be larger than the amount of vitellogenin (Band 1) in wasp extract. Preincubation of the sera with a CCD‐inhibitor did not affect the observed IgE reactivity (binding of Pool 1 to wasp venom proteins, Figure 4E).

### 3.5 Putative cross‐reactive peptides were identified

Using an *in silico* approach, we predicted possible cross‐reactive peptides by investigating sequence similarity between tick hemelipoglycoprotein and wasp vitellogenin (G8IIT0). A total of 17 peptide combinations was considered to be possible cross‐reactive peptides due to their PD value of <7 (Table 2). A PD‐value of 5.34 was the lowest PD‐value detected.

**TABLE 2:** Peptides similarity between tick hemelipoglycoprotein (A0A0D3RJ94) and wasp vitellogenin (G8IIT0) based on *in silico* analysis

Proteins	Amino acids	Position	PD‐value^a^
Tick hemelipoglycoprotein (A0A0D3RJ94)	DQSSITFKGK	1177–1186	5.37
Wasp vitellogenin (G8IIT0)	DNSLVTIKGQ	1274–1283	
Tick hemelipoglycoprotein (A0A0D3RJ94)	VDGNRVQLTQ	1432–1441	5.67
Wasp vitellogenin (G8IIT0)	VNGQKVKCSQ	1517–1526	
Tick hemelipoglycoprotein (A0A0D3RJ94)	VYEQAVANAP	367–376	5.78
Wasp vitellogenin (G8IIT0)	VFRDAIANAG	497–506	
Tick hemelipoglycoprotein (A0A0D3RJ94)	ANTDTQLPDD	268–277	5.90
Wasp vitellogenin (G8IIT0)	GNKNTQIPED	154–163	
Tick hemelipoglycoprotein (A0A0D3RJ94)	SVFAQVRADD	676–685	6.33
Wasp vitellogenin (G8IIT0)	SIISQFQADT	138–147	
Tick hemelipoglycoprotein (A0A0D3RJ94)	TKIKNLEKCD	178–187	6.50
Wasp vitellogenin (G8IIT0)	TKTRNYDKCE	217–226	
Tick hemelipoglycoprotein (A0A0D3RJ94)	DDEAEHFLTK	414–423	6.63
Wasp vitellogenin (G8IIT0)	NNEAETFDGK	1435–1444	
Tick hemelipoglycoprotein (A0A0D3RJ94)	YVTSAFRSLV	613–622	6.63
Wasp vitellogenin (G8IIT0)	QVNSAVKSAI	732–741	
Tick hemelipoglycoprotein (A0A0D3RJ94)	ELRYSFTKDN	1102–1111	6.68
Wasp vitellogenin (G8IIT0)	EIDMTITKHN	1326–1335	
Tick hemelipoglycoprotein (A0A0D3RJ94)	LTDDEAEHFL	412–421	6.69
Wasp vitellogenin (G8IIT0)	LRNNEAETFD	1433–1442	
Tick hemelipoglycoprotein (A0A0D3RJ94)	RPFNQGKTFV	244–253	6.70
Wasp vitellogenin (G8IIT0)	KGINSGKAYV	1176–1185	
Tick hemelipoglycoprotein (A0A0D3RJ94)	NVFRPFNQGK	241–250	6.73
Wasp vitellogenin (G8IIT0)	EVVKGINSGK	1173–1182	
Tick hemelipoglycoprotein (A0A0D3RJ94)	QVWVNCQLAL	451–460	6.78
Wasp vitellogenin (G8IIT0)	QVFLPCKLDF	990–999	
Tick hemelipoglycoprotein (A0A0D3RJ94)	ESILQELSKG	790–799	6.80
Wasp vitellogenin (G8IIT0)	KEFLQEVVKG	1168–1177	
Tick hemelipoglycoprotein (A0A0D3RJ94)	VPSELGVPVF	832–841	6.84
Wasp vitellogenin (G8IIT0)	FPTETGLPFV	924–933	
Tick hemelipoglycoprotein (A0A0D3RJ94)	DEAEHFLTKL	415–424	6.92
Wasp vitellogenin (G8IIT0)	NEAETFDGKV	1436–1445	
Tick hemelipoglycoprotein (A0A0D3RJ94)	KRKKSFILSK	817–826	6.93
Wasp vitellogenin (G8IIT0)	DDQESIVISK	288–297	

Abbreviation: PD, property distance.

^a^ All peptides with a PD value < 7.0 has been listed.

*Source*: Performed by Structural Database of Allergenic Proteins (SDAP, http://Fermi.utmb.edu/SDAP/).

## 4 DISCUSSION

We investigated the role of wasp sensitization in Swedish AGS patients and for the first time described cross‐reactivity between tick and wasp. We found that IgE reactivity to wasp was frequent in AGS patients from Sweden, although the IgE levels were in general low. Wasp sensitized patients were found to have higher IgE levels to α‐Gal and total IgE levels compared to non‐wasp sensitized AGS patients. α‐Gal could not be detected on wasp venom proteins, but we observed cross‐reactivity between wasp and tick proteins, which may be one explanation for the higher percentage of wasp sensitization in tick bitten AGS patients compared to the general population. The same cross‐reactivity was also observed in wasp‐positive non‐AGS control sera.

Fifty‐four percent of the Swedish AGS patients were sensitized to wasp, which is high compared to the general population. The percentage of wasp sensitization in Europe ranges from 9% to 30%, depending on climate.^12^ In colder areas like Sweden and Denmark, only 9% and 15% of the population have been reported to be sensitized to wasp respectively.^27^, ^28^ These data are in line with a study on wasp sensitization in AGS patients, which states that AGS patients from the US have a five times higher risk of wasp sensitization compared to healthy controls.^8^

In our cohort of AGS patients, wasp sensitization was found to be associated with higher α‐Gal IgE levels. We found no evidence for the presence of α‐Gal bearing proteins in wasp venom, which makes it unlikely that wasp stings are directly involved in α‐Gal sensitization. However, this finding can be related to the amount of time people spend outdoors. Patients who spend much time in nature have a higher risk of having higher levels of α‐Gal due to multiple tick bites,^9^ as well as of wasp sensitization.^11^, ^12^ The occurrence of co‐sensitization is indeed confirmed by the presence of Ves v 5‐specific IgE in the AGS patient serum pool.

Furthermore, we observed that the total IgE levels were higher in wasp‐sensitized AGS patients compared to non‐wasp sensitized AGS patients, which is in line with previous data from the general population.^29^ Additional immunological factors like atopy have shown to predispose an individual to α‐Gal sensitization and the development of AGS,^14^ as well as to wasp sensitization.^13^ The question whether other immunological predispositions might be involved in co‐sensitization to wasp and tick has been raised before,^8^ but no data is available so far. Thus, more detailed studies on the immune response in the skin after ecto‐parasite bites and insect stings would be valuable to better understand the current observations.

In this study, we show for the first time cross‐reactivity between tick‐ and wasp‐specific IgE. This may be one explanation for the high number of wasp‐sensitized AGS patients. Tick protein was found to inhibit the binding of wasp‐IgE in an AGS serum pool, in individual AGS patient sera as well as in wasp‐positive control sera. Using WBs we found the lipid transfer protein hemelipoglycoprotein (∼290 kDa) as the cross‐reacting protein in tick extract, which has previously been detected in *I. ricinus* by mass spectrometry,^2^ and in many other *Ixodes* tick species.^22^ Hemelipoglycoprotein is structurally and functionally closely related to tick vitellogenin.^30^ They show a sequence similarity of 97%, and previous studies only differentiated the proteins by their expression profiles.^30^, ^31^ Vitellogenin has been identified to be the cross‐reactive protein in wasp venoms. Early research has already described this protein in the *Pimpla nipponica* wasp,^24^ which was reported as a new wasp allergen (Ves v 6) in 2013.^23^ Based on the SDS‐PAGE, we concluded that the amount of hemelipoglycoprotein in tick extract is larger than the amount of vitellogenin in wasp extract, which explains why wasp venom did not exert a strong inhibition of tick IgE binding. Another explanation could be that tick bite induced IgE antibodies have a higher specificity towards tick hemelipoglycoprotein than to wasp vitellogenin (G8IIT0).

Vitellogenin is an egg yolk precursor which is present in almost all female oviparous animals. It has been recognized as a pan‐allergen in different mite species,^32^ shrimp,^33^ and insects like cockroach,^34^ bee and wasp,^23^ and also in ticks.^2^ This leads to possible cross‐reactivity between these species,^35^ which is in line with our finding. The clinical relevance of IgE against vitellogenin is still unknown. However, the fact that a substantial proportion of different investigated populations show IgE reactivity to vitellogenin from different sources suggests their potential importance. For example, 67% of atopic dermatitis patients recognized the vitellogenin‐like protein Der p 14,^36^ 47% of cockroach allergic patients recognized Bla g vitellogenin,^34^ and 40% of venom‐sensitized individuals recognized the vitellogenin Ves v 6.^23^

IgE reactivity to CCDs is notorious for causing cross‐reactivity in plants and insects.^37^ However, wasp vitellogenin (G8IIT0) has previously been demonstrated to cause sensitization in venom allergic patients independent of its carbohydrate structures.^23^ We did not observe any effect of a commercial CCD inhibitor on the IgE reactivity of the tick‐positive serum pool against wasp protein. Since we have earlier reported that only a few of our AGS patients show IgE‐reactivity against MUXF,^38^ our results point to the fact that the observed cross‐reactivity between tick and wasp is not due to CCD reactivity, but rather to similarities in protein structures.

We noted that the majority of our random population of wasp‐positive sera was solely wasp‐IgE positive. These sera showed only binding to Ves v 5 and Ves v 1, which were not inhibited by tick protein extract (Figure 4C). However, in the wasp‐ and tick‐positive control serum pool we observed the same cross‐reactivity due to vitellogenin as in AGS patients (Figure 4D). The results highlight that low IgE levels to wasp due to tick sensitization, might be an overlooked issue in individuals in areas with a high tick density, like Sweden, because tick IgE can so far not be commercially determined.

Although vitellogenin has been described as an allergen in multiple species, the exact allergen epitopes have not been identified yet, and it is not known which part of the protein is cross‐reactive with vitellogenin and vitellogenin‐like proteins from other species. Sequence similarity assessment including the whole tick hemelipoglycoprotein and wasp vitellogenin (G8IIT0) revealed a list of 17 possible cross‐reactive peptides. This *in silico* analysis confirms our experimental data by showing that cross‐reactivity is likely based on the primary structure of the proteins, and provides candidate peptides for further research focusing on the identification of the allergen epitopes in vitellogenin.

In conclusion, for the first time cross‐reactivity between wasp venom and tick has been described. This may be one reason why more than half of Swedish AGS patients, who have all been tick bitten, are also sensitized against wasp, in addition to other factors, for example, environmental factors. Furthermore, the clinical relevance of the observed cross‐reactivity between tick and wasp proteins needs further research.

## CONFLICT OF INTEREST

Dr. van Hage reports personal fees from Thermo Fisher Scientific, outside the submitted work. Dr. Starkhammar reports fees from Mylan, ALK, AstraZeneca and Chies. The rest of the authors declare no conflict of interest.

## Supporting information

Supporting Information S1
