# Supplementary material for: Cross‐reactivity between tick and wasp venom can contribute to frequent wasp sensitization in patients with the α‐Gal syndrome
Source: Clin Transl Allergy. 2022 Jan 17;12(1):e12113. doi: 10.1002/clt2.12113 (PMC8762686; doi:10.1002/clt2.12113)
Supplement: Supplementary file 1 — Supporting Information S1 [file CLT2-12-e12113-s001.docx]

Additional file

**Cross-reactivity between tick and wasp venom can contribute to frequent wasp sensitization in patients with the α-Gal syndrome.**

M B Gea Kiewiet^1^, PhD; Marija Perusko^1^, PhD; Jeanette Grundström^1^, PhD; Carl Hamsten^1^, PhD; Maria Starkhammar^2^, MD; Danijela Apostolovic^1^, PhD; Marianne van Hage^1^, MD, PhD.

1. Department of Medicine Solna, Division of Immunology and Allergy, Karolinska Institutet and University Hospital, Stockholm, Sweden;

2. Department of Internal Medicine, Södersjukhuset, Stockholm, Sweden.

Table S1. IgE levels to wasp, tick and α-Gal of sera from wasp-positive controls.

| Serum | Wasp IgE (kU_A_/L) | Tick IgE (kU_A_/L) | α-Gal IgE (kU_A_/L) |
| --- | --- | --- | --- |
| *1* | 0.67 | <0.10 | <0.10 |
| *2* | 12.0 | <0.10 | <0.10 |
| *3* | 1.10 | 20.0 | <0.10 |
| *4* | 7.50 | <0.10 | <0.10 |
| *5* | 1.60 | <0.10 | <0.10 |
| *6* | 0.73 | <0.10 | <0.10 |
| *7* | 0.54 | <0.10 | <0.10 |
| *8* | 66.0 | <0.10 | <0.10 |
| *9* | 4.80 | <0.10 | <0.10 |
| *10* | 0.90 | <0.10 | <0.10 |
| *11* | 0.60 | <0.10 | <0.10 |
| *12* | 0.61 | 1.50 | 0.30 |
| *13* | 6.80 | <0.10 | <0.10 |
| *14* | 0.48 | <0.10 | <0.10 |
| *15* | 0.69 | <0.10 | <0.10 |
| *16* | 1.00 | <0.10 | <0.10 |
| *17* | 2.90 | <0.10 | <0.10 |
| *18* | 0.70 | 3.30 | <0.10 |
| *19* | 0.80 | <0.10 | <0.10 |
| *20* | >100 | 13.0 | <0.10 |
| *21* | 5.30 | 0.72 | <0.10 |
| *22* | 24.0 | 0.27 | 1.30 |
| *23* | 7.00 | <0.10 | <0.10 |
| *24* | 9.20 | <0.10 | <0.10 |
| *25* | 9.00 | 0.16 | 0.85 |
| *26* | 7.50 | 0.79 | 0.47 |
| *27* | 80.0 | <0.10 | 0.14 |
| *28* | 13.0 | <0.10 | <0.10 |
| *29* | 26.0 | <0.10 | <0.10 |


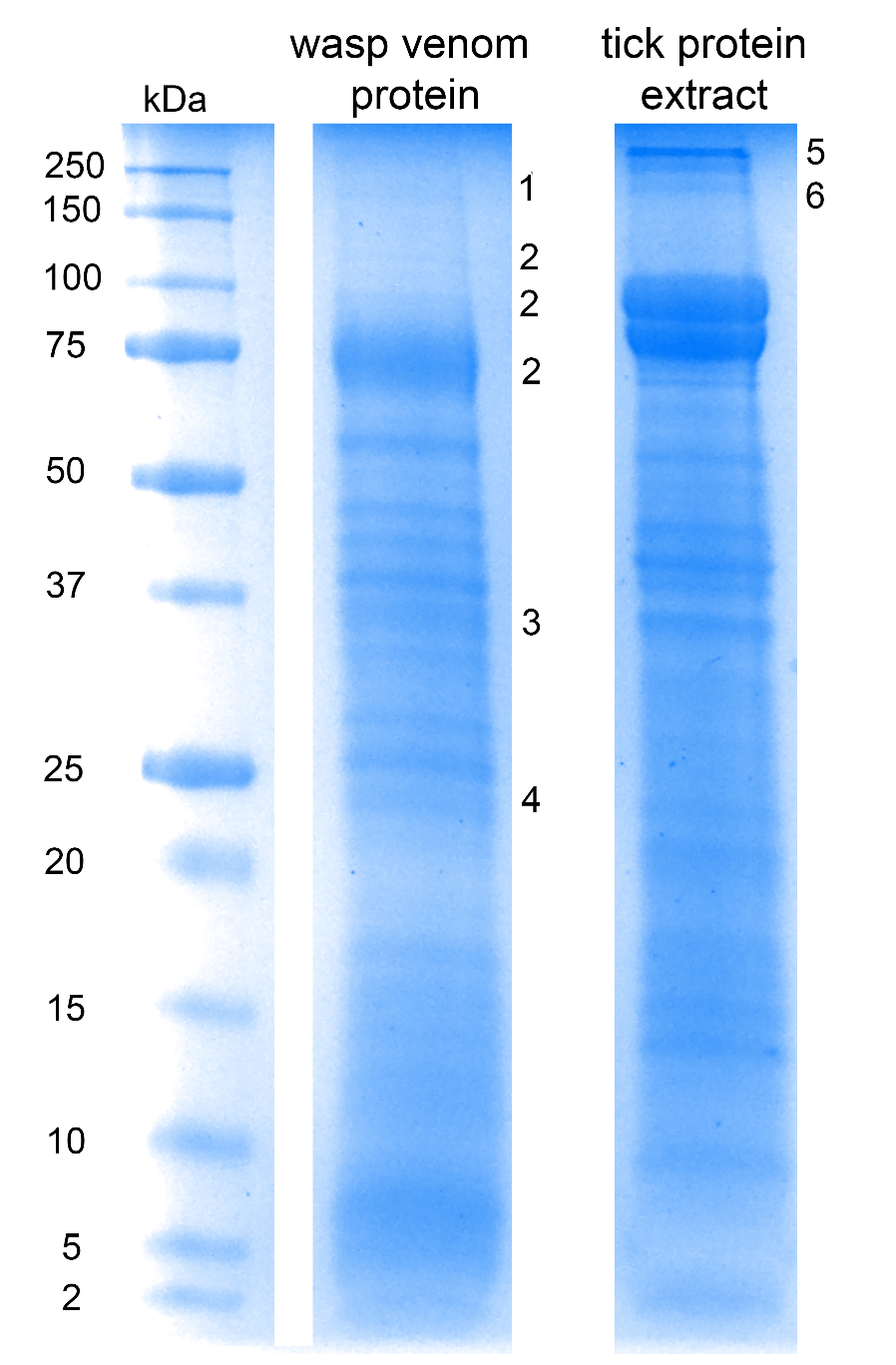


## Figure S1. Coomassie Brilliant Blue (CBB) staining of SDS-PAGE protein patterns of wasp venom protein and tick protein extract (*I. ricinus*). 1=vitellogenin, 2= vitellogenin subunits, 3= Ves v 1, 4= Ves v 5, 5= hemelipoglycoprotein, 6= hemelipoglycoprotein subunit.
